# Free Will, Moral Responsibility, and Scientific Epiphenomenalism

**DOI:** 10.3389/fpsyg.2018.02536

**Published:** 2018-12-11

**Authors:** Alfred R. Mele

**Affiliations:** Department of Philosophy, Florida State University, Tallahassee, FL, United States

**Keywords:** causation, consciousness, epiphenomenalism, free will, intentions, Libet, moral responsibility, Wegner

## Abstract

This article addresses two influential lines of argument for what might be termed “scientific epiphenomenalism” about conscious intentions – the thesis that neither conscious intentions nor their physical correlates are among the causes of bodily motions – and links this thesis to skepticism about free will and moral responsibility. One line of argument is based on Benjamin Libet’s neuroscientific work on free will. The other is based on a mixed bag of findings presented by social psychologist Daniel Wegner. It is argued that both lines of argument are unsuccessful.

In an influential book, *The Illusion of Conscious Will*, Daniel Wegner writes:

The experience of consciously willing an action... serves as a kind of compass, alerting the conscious mind when actions occur that are likely to be the result of one’s own agency. The experience of will is therefore an indicator, one of those gages on the control panel to which we refer as we steer. Like a compass reading, the feeling of doing tells us something about the operation of the ship. But also like a compass reading, this information must be understood as a conscious experience, a candidate for the dreaded “epiphenomenon” label. Just as compass readings do not steer the boat, conscious experiences of will do not cause human actions.

This chapter examines why the conscious experience of will might exist at all. Why, if this experience of will is not the cause of action, would we even go to the trouble of having it? What good is an epiphenomenon? (2002, pp. 317–318)

As I will briefly explain in section 1, Wegner’s use of “epiphenomenon” here diverges from standard philosophical usage. Even so, in this passage he places himself in the ballpark of this journal issue’s topic – “The New Science of Free Will: The Epiphenomenalist Challenge.”

Wegner (2002, 2004, 2008) maintains that conscious intentions and decisions are never among the causes of corresponding actions and he uses two lines of argumentation to support his thesis. One line is based on Benjamin Libet’s influential neuroscientific work, which I discuss in section 2. The other is based on a mixed bag of findings that I discuss in section 3. Section 1 provides background, and section 4 wraps things up.

## Background

Brief attention to some key notions will prove useful. I start with deciding. Deciding to do something, as I understand it, is a momentary action of forming an intention to do it (Mele, 2003, ch. 9). Reasoning about what to do typically is not momentary, but it must be distinguished from an act of deciding that is based on reasoning. Some decisions and intentions are about things to do right away. They are *proximal* decisions and intentions. Others – *distal* decisions and intentions – are about things to do later. I might decide now to answer the knock at my door now. This is a proximal decision. And I might decide now to write a blurb for a friend’s book tomorrow. This is a distal decision. Libet’s work on decisions and intentions focuses on the proximal variety. The same is true of Wegner.

Decisions about what to do, as I understand them, are responses to uncertainty about what to do (Mele, 2003, ch. 9). Where there is no uncertainty, we make no decisions. Because I was not at all uncertain about what to do when I got to my office door this morning, I had no need to decide what to do when I arrived at the door. I simply unlocked it, as I intended to do. That intention was passively acquired, as opposed to being actively formed in an act of deciding to unlock my door. By “passively acquired” I mean nothing more than “acquired in the absence of any action of forming that intention.” Both actively formed and passively acquired intentions are suitable candidates for issuing in corresponding intentional actions.

A comment on the adjective “intentional” will prove useful in avoiding some possible misinterpretations of what is to come. Compare the task of flexing one’s right wrist whenever one wishes with the task of flexing one’s right wrist whenever one hears a go-signal. As I use the adjective “intentional,” both tasks involve intentional flexing actions. But as some researchers use the adjective, the flexing actions of the latter group, unlike those of the former group, are not intentional (see Haggard and Clark, 2003, pp. 695–696, and see the discussion of their usage and mine in Mele, 2009, pp. 29–30). They count “self-initiated,” but not “externally triggered,” actions as intentional. As I see things, these researchers use “intentional” as a technical term whereas I use it as it is used in ordinary speech. I do not object to the use of ordinary-language terms as technical terms. However, readers accustomed to the technical use of “intentional” at issue, unless forewarned, may misinterpret some of what I have to say below about intentional actions and about associated intentions.

Consider the following passage:

Filomena is stopped at a red light in an old car that she has been driving for many years. When the light turns green, she presses down on her gas pedal. She doesn’t know it, but there’s a problem with her engine. By pressing on the gas pedal, Filomena releases a lot of exhaust fumes into the air and irritates the cyclist behind her.

As I use the key terms, Filomena *intends* to press down on her gas pedal and *intentionally* does so (but she does not intentionally release a lot of exhaust fumes and does not intentionally irritate the cyclist; nor does she intend to do either of these things). Filomena’s pressing down on the gas pedal is an externally triggered action. It is triggered by her detection of an everyday go-signal – the light turning green. And, even so, I count it as intentional. Have I gone out on a limb in claiming that I am simply following ordinary usage? Not at all. The passage about Filomena is one of nine test cases in a study that Thomas Nadelhoffer and I very recently completed for another purpose (too recently to have published it yet). We asked the 202 MTurk participants whether they agreed or disagreed with various statements about the vignettes, using a seven-point scale (ranging from “strongly agree” to “strongly disagree”). Here are results for two of the statements about Filomena, counting answers above the midpoint as expressing agreement and answers below the midpoint as expressing disagreement:

Filomena intentionally pressed down on her gas pedal. Agree 96.52%. When she pressed down on her gas pedal, Filomena intended to do that. Agree 94.05%^1^.

Readers who have not adopted a technical use of “intentional” and “intentionally” may think that I have been belaboring an obvious point. But some readers are accustomed to the technical use of “intentional” described above. The preceding two paragraphs are addressed to them. My intent is to forestall confusion about some subsequent claims of mine.

Next on the agenda is epiphenomenalism. What I will call *philosophical epiphenomenalism* (and what philosophers refer to simply as *epiphenomenalism*) is the thesis that although all mental events are caused by physical events, no mental events are among the causes of any physical events. Some scientists appeal to findings of the kind reviewed below to support the idea that conscious decisions and intentions are what they call “epiphenomena” (Wegner, 2002, pp. 317–318). However, as I will explain, what they mean by this word in this connection is not always what philosophers mean by it. Bearing this point in mind helps forestall confusion.

If, as Wegner (2002, p. 317) says, “the experience of consciously willing an action” – or a conscious intention one has, or consciousness of an intention one has – alerts “the conscious mind when actions occur that are likely to be the result of one’s own agency,” then the conscious event or state that plays the alerting role is among the causes of something. Why so? Because alerting is a causal notion. If something alerts me to some fact, it has an *effect* on me. It is among the causes of a change in me, a change from being unaware of the fact at issue to being aware of it. Now, suppose I am asked what I am about to do, and I orally report what I believe about that. Suppose the report is based on what my consciousness of an intention of mine alerted me to. My utterance (about what I believe I am about to do) is a physical event (or has a physical dimension), and my consciousness of my intention is, by hypothesis, among its causes. So my consciousness of my intention is not an epiphenomenon, as that notion is characterized by philosophical epiphenomenalism. Even so, Wegner calls it an epiphenomenon. We see right off the bat that he means something else by the term. This is not intended as a criticism. It is a way of setting the stage for Wegner’s intended meaning, which is the next item on the agenda.

As a convenient bit of shorthand, I use “proximal intentions+” as a label for the following collection of things: proximal intentions themselves, their acquisition, and their persistence. Proximal intentions+, then, include my intention to answer my ringing phone now, my acquiring that intention (when I hear the phone ring), and the persistence of that intention (until I succeed in pulling the phone out of my pocket and greeting the caller). Consider the following two hypotheses: First, all proximal intentions+ are caused by physical events but no proximal intentions+ are among the causes of any physical events. Second, physical correlates of proximal intentions+ sometimes *are* among the causes of physical events – for example, bodily motions involved in overt intentional actions. Although this pair of hypotheses does not contradict philosophical epiphenomenalism, it does contradict a scientific epiphenomenalism according to which neither proximal intentions+ nor their physical correlates are among the causes of bodily motions (Wegner, 2002, 2004, 2008). The scientific epiphenomenalism at issue in this article extends to the physical correlates of proximal intentions+: the thesis to be examined – Wegner’s thesis, as I understand him – is that neither proximal intentions+ nor their physical correlates are among the causes of physical events that proximal intentions+ are alleged to cause, those involved in corresponding overt intentional actions.

Philosophical epiphenomenalism is one of the options on the table in philosophical work on mental causation. This work addresses a very old and very deep topic – the mind-body problem. We have a mental vocabulary – for example, “decision,” “intention,” and “desire” – and a physical vocabulary: “neuron,” “synapse,” and “brain” come quickly to mind in the present context. An important question in metaphysics and in the philosophy of mind is easy to state and hard to answer: How are the things to which these two vocabularies refer related to each other? The following question is more specific: How are mental processes and events – such as my reflection on what topics to take up in this article and my decision on the matter – related to physical processes and events? Neither of these questions is directly about mental causation, but the following questions are: Does our conscious reflection about what to do ever make a causal contribution to what we do? (Less formally: Does our conscious reflection ever have an effect on what we do?) Are our decisions about what to do ever among the causes of corresponding actions? (For example, was my decision to accept the invitation I received to contribute to this journal issue among the causes of my sending an e-mail message reporting my acceptance?)

How one answers the first two questions raised in the preceding paragraph may have an effect on how one answers the questions about mental causation. Consider substance dualism. This position features an endorsement of the idea that “associated with each human person, there is a thinking thing... not composed of the same kinds of stuff as... nonmental things” (Zimmerman, 2006, p. 115; although Zimmerman describes the “thinking thing” as a soul, some substance dualists prefer to use the word “mind”). Someone who thinks that conscious reflection takes place only in non-physical souls or minds and that conscious decisions are made only by such souls or minds may be stumped by the question how such reflection and decisions can have an effect on the body – for example, on the motions of my fingers as I type this sentence.

Fortunately, there are philosophical alternatives to substance dualism. Conscious intentions might, for example, be *identical to* states of the brain, or they might be *realized in* or *supervene on* brain states. Identity is relatively easy to understand. Supervenience is more complicated, and various kinds have been distinguished. ^2^ Here is David Papineau’s (2009, p. 62) gloss on one kind: for non-physical (including mental) properties to “*metaphysically supervene* on” physical properties is for things to be such that “a being’s physical properties... fix its non-physical properties, in the sense that any two [hypothetical] beings who share [all the same] physical properties... necessarily share the same non-physical properties, even though the physical properties which so “realize” the non-physical ones can be different in different beings”. Papineau adds: “This arguably ensures that nothing more is required for any specific instantiation of a non-physical property than its physical realization.” The basic idea here, as it applies specifically to me and my conscious intention to complete this paragraph before leaving for lunch, is (1) that my being in a certain physical condition is sufficient for my having that conscious intention, and (2) my being in some other physical condition might also have been sufficient for my having that conscious intention.

It is a rare scientist who also is a metaphysician; and, generally speaking, scientists have no obligation to weigh in on metaphysical connections between mental states and physical states – for example, on whether conscious intentions are identical with brain states or instead supervene on them. From a scientific perspective, as I observed elsewhere, “evidence that the physical correlates of conscious intentions are among the causes of some corresponding actions may be counted as evidence that conscious intentions are among the causes of some corresponding actions, and evidence that the physical correlates of conscious intentions are never among the causes of corresponding actions may be counted as evidence that conscious intentions are never among the causes of corresponding actions” (Mele, 2013a, p. 9). In this connection, I invited readers to “try to imagine a *scientific* discovery that the physical correlates of conscious intentions actually *are* (or actually are not) conscious intentions or that conscious intentions do (or do not) supervene on their physical correlates” (ibid). And I raised the following pair of pointed questions. How would the discovery be made? What would the experimental design be? As I pointed out in Mele, 2009 (p. 146), it is primarily philosophers who would worry about the metaphysical intricacies of the mind-body problem despite accepting the imagined proof about physical correlates of conscious intentions, and the argumentation would be philosophical in nature.^3^

Another issue that cannot be settled scientifically is whether the following proposition is true or false: The physical correlates of conscious intentions (that is, of the intentions *and* consciousness of them) sometimes are among the causes of corresponding actions, but conscious intentions never are. Why not? Because (even if it can be done in imagination) there is no way for scientists to separate the physical correlates of conscious intentions from the conscious intentions to see what each does on its own. Whether the proposition at issue is true or false is a question for metaphysicians and is well beyond the scope of the present article. My topic is *scientific* arguments for (scientific) epiphenomenalism.

In subsequent sections, I discuss findings in neuroscience and elsewhere that have been thought to bear significantly on mental causation in the sphere of free will and moral responsibility. An additional bit of background will prove useful.

In the philosophical literature on free will and moral responsibility, it is commonly assumed that the latter depends on the former. The assumption is not that the only actions for which we can be morally responsible are actions that we perform freely or of our own free will. Seemingly, drunk drivers can be morally responsible for losing control of their cars and killing people as a consequence, even if they do not do these things of their own free will. Rather, what is assumed is that a being who never has free will is not morally responsible for anything.^4^ When this assumption is in place, any argument for the nonexistence of free will is an argument for the nonexistence of moral responsibility.

## Libet

In their introduction to an edited volume entitled *Does Consciousness Cause Behavior?* Susan Pockett and her coeditors write: “the wide promulgation of two new lines of genuinely scientific... evidence has seized the philosophical and scientific imagination and again brought the whole question [whether consciousness causes behavior] to the forefront of intellectual debate” (Pockett et al., 2006, p. 1). They identify neuroscientist Benjamin Libet and social psychologist Daniel Wegner as the sources of these lines of evidence (pp. 1–3). According to Libet (1985, 2004), the brain decides to initiate actions about a third of a second before the person becomes aware of the decision. Wegner (2002, 2004, 2008) contends, as I have mentioned, that conscious intentions and decisions are never among the causes of corresponding actions.

Libet makes the following much-discussed claims:

The brain “decides” to initiate or, at least, prepare to initiate [certain actions] before there is any reportable subjective awareness that such a decision has taken place. (Libet, 1985, p. 536)

If the “act now” process is initiated unconsciously, then conscious free will is not doing it. (Libet, 2001, p. 62)

Our overall findings do suggest some fundamental characteristics of the simpler acts that may be applicable to all consciously intended acts and even to responsibility and free will. (Libet, 1985, p. 563)

Associated with these claims is a skeptical argument about free will that may be set out as follows.

*F1*. In Libet-style experiments, all the decisions to act on which data are gathered are made unconsciously.

*F2*. So probably all decisions to act are made unconsciously.

*F3*. A decision is freely made only if it is consciously made.

*F4*. So probably no decisions to act are freely made^5^.

Participants in the studies at issue are asked to report on when they had certain conscious experiences – described variously as experiences of an urge, intention, or decision to do what they did. They make their reports after they act. In what follows, readers who understand “conscious” and “aware” in such a way that one can be aware of something of which one is not conscious (on the grounds that consciousness requires a phenomenal feature that awareness does not) should read “conscious” as “aware.” (More simply, for the purposes of this article, I treat “conscious of” and “aware of” as synonyms.) Also, the measure of consciousness or awareness in these studies is the participant’s report. As I have put it (Mele, 2009, p. 22), it is “report-level” consciousness or awareness that is at issue.

In Libet’s (1985, 2004) most influential studies, participants are asked to flex their right wrist whenever they wish. When they are regularly reminded not to plan their wrist flexes, an average ramping up of EEG activity (starting 550 ms before the onset of muscle motion; that is, -550 ms) precedes the average reported time of the conscious experience (-200 ms) by about a third of a second (Libet, 1985). Libet (1985, p. 536) contends that decisions about when to flex are made at the earlier of these two times. The later time is referred to as *W time*.

The initial ramping that I mentioned is the beginning of a readiness potential (RP), which may be understood as “a progressive increase in brain activity prior to intentional actions, normally measured using EEG, and thought to arise from frontal brain areas that prepare actions” (Haggard et al., 2015, p. 325). The RPs I described are called type II RPs. Participants who are not regularly reminded to aim for spontaneity or who report some advance planning produce RPs that begin about half a second earlier – type I RPs. This also is true of participants asked to flex at a prearranged time (Libet et al., 1982, p. 325).

Elsewhere, I have argued in detail for the following points about *F1*. (1) There is no good reason to believe that a proximal decision to flex is made – or a proximal intention to flex acquired – at -550 ms, and there is evidence that any such decision or intention arrives on the scene significantly later, around W time (Mele, 2009, chs. 3 and 4). (2) There is good reason to doubt that the W times are reliable guides to when participants were, in fact, first conscious of their intentions or decisions (Mele, 2009, ch. 6). In order to avoid excessive repetition, I do not rehearse these arguments here. Instead, I focus my critique on *F2*.

When moral responsibility is at issue, as it is in this article, an elementary observation about *F2* should set off an alarm. Obviously, how plausible the inference reported in *F2* is depends on how similar decisions to act made by participants in Libet-style studies are to decisions to act of all other kinds. So how similar are these decisions to ordinary decisions about what to do when moral considerations are in play, for example? (I assume for the sake of argument that participants do make decisions about when to flex, and I believe that this is a genuine possibility.)

Participants in Libet’s studies apparently arbitrarily pick a moment to begin flexing – many times. Arbitrary picking is characteristic of neuroscientific studies of free will. Participants are supposed to decide when to flex a wrist, which of two buttons to press (Soon et al., 2008), when to press a key (Fried et al., 2011), and so on. They have no reason to prefer a particular moment for beginning to flex a wrist or press a key over nearby moments and no reason to prefer one button over the other. They are in situations in which arbitrary picking makes perfect sense. As I have implied, I am assuming that these instances of arbitrary picking are instances of deciding. I do not treat arbitrary picking and deciding as mutually exclusive.

Here, we find one difference between decisions allegedly made in the experiments at issue and many other decisions. In these experiments, participants select from options they are indifferent about. However, in many cases, decision makers are far from indifferent about their leading options. In typical cases, when we make a decision about whether to accept or reject a job offer, whether or not to make a bid on a certain house, and so on, our leading options differ from one another in ways that are important to us. The same is true of decision making when moral considerations are explicitly in play. People who are tempted to cheat on their taxes normally are not indifferent between cheating on them and being honest. Decisions about such things as evading taxes and whether or not to lie in order to get out of a jam do not seem much like arbitrary picking. Often, both when moral considerations are salient and when they are not, we are not indifferent about what we decide.

Another difference between decisions allegedly made in Libet-style experiments and some other decisions has to do with conscious reasoning. In these experiments, conscious reasoning about what to do – for instance, about exactly when to flex – is rendered pointless by the nature of the task. As I have mentioned, the task is such that there obviously is no reason to prefer a given moment for flexing to nearby moments, no reason to prefer pressing one button to pressing the other, and so on. So reasoning about when to flex, which button to press, or the like, would be pointless. Furthermore, in these studies, participants are instructed to be spontaneous, which involves not reasoning about what to do. However, many decisions are preceded by conscious reasoning about what to do. Elsewhere, I have suggested that such reasoning may increase the probability of conscious deciding, where consciously deciding to do something is understood as a matter of being conscious of the decision *when* one makes it – as opposed, for example, to becoming conscious of the decision after one has made it (Mele, 2013b).

The alleged findings about decisions in scenarios in which, as the participants realize, they have no reason to favor any acceptable option over any other do not warrant the conclusion that the same kind of thing would be found in cases in which decision makers are not indifferent about their options and are not arbitrarily picking something. As I have suggested elsewhere, automatic tie-breaking mechanisms may be at work in a variety of ordinary situations in which we are indifferent regarding the leading options: for example, when I have a twenty-six ounce of Campbell’s tomato soup on my shopping list and I see a bunch of them on a shelf in my favorite supermarket (Mele, 2009, p. 83). And there is no good reason to believe that what happens in cases of indifference is also what happens when unsettledness about what to do leads to careful conscious reasoning about what to do. Some action-ties may be broken for us before we are aware of what we “decided” to do, but this definitely does not justify the claim that we never consciously make decisions.

As I reported, I assume for the sake of argument that participants in Libet’s experiments do make proximal decisions and I regard this as a genuine possibility. My point is that the circumstances surrounding these arbitrary decisions are so different from the circumstances surrounding many decisions that we cannot properly generalize from the alleged finding that the former decisions are made unconsciously to the conclusion that all decisions are made unconsciously. Suppose a study of Donald Trump’s golf courses reveals that all of them prominently display a painting of Trump decked out in a blue jacket and a long red tie. No reasonable person would infer from this that all golf courses – no matter who owns them – display a painting of Donald Trump. On formal grounds, the generalization at issue about decisions is almost as bad.

Here is the bottom line on *F2*. A generalization from alleged findings about the decisions allegedly made in Libet-style studies to the claim that all decisions are unconsciously made is unwarranted.

Some readers who agree with my bottom line on *F2* may ask whether, at least in Libet-style studies, proximal decisions are made unconsciously. Given space constraints, the best I can do here in response is to point such readers to my arguments elsewhere that the data do not support this proposition about participants in these studies (Mele, 2009, chs. 3, 4, and 6).

It is sometimes claimed, based partly on Libet’s findings, that conscious intentions and decisions are caused by the same brain events that cause actions and are absent from the causal chain that issues in action (Wegner, 2002, pp. 64–70). The following assertion sometimes is offered in support of the preceding one: Participants’ conscious proximal intentions to flex cannot be among the causes of their flexes because those intentions are caused by unconscious brain events (Pockett, 2006, p. 21; Roediger et al., 2008, p. 208). This assertion about mental causation is seriously confused (and, as a referee motivated me to add, I certainly am not claiming that many scientists do or would make it). The confusion is easy to see with the help of an analogy (see Mele, 2009, p. 71). Consider the following claim: Burnings of fuses cannot be among the causes of explosions of firecrackers because burnings of fuses are caused by lightings of fuses. Plainly, both the lighting of its fuse and the burning of its fuse are among the causes of a firecracker’s exploding in ordinary situations. If the fuse had not been lit – or if the lit fuse had stopped burning early – there would have been no explosion. Just as there is no reason to believe that the more proximal causes of firecracker explosions cannot themselves have causes, there is no reason to believe that items that are among the relatively proximal causes of flexes cannot themselves have causes and cannot be caused by unconscious brain events. That a conscious decision or intention has unconscious causes is no obstacle to the decision’s or intention’s being among the causes of a corresponding action. And if some decisions or intentions (or their physical correlates) are among the causes of corresponding actions, scientific epiphenomenalism is false.

## Wegner

As I mentioned, Wegner uses two lines of argumentation to support his claim that conscious intentions are never among the causes of corresponding actions, one of which is based on Libet’s work. I turn now to the second line, starting with some findings.

Facilitated communication is a technique designed to help people who have disorders that hamper speech, such as autism or cerebral palsy, express themselves (Wegner, 2002, pp. 195–201). The facilitator holds the hand of a client seated in front of a keyboard. The facilitator’s function is to help clients communicate without influencing which keys they press, and there is evidence that many of the facilitators believed they were doing exactly what their job called for. Often, formerly uncommunicative people apparently typed full sentences or more. It was discovered, however, that the facilitators were controlling what was being typed – without realizing that they were. They were unconsciously controlling their clients’ movements.

Sensitive devices can detect some actions that people do not realize they are performing (Wegner, 2002, pp. 122–25). For example, a person instructed to think of an object located to his left slowly moved a hand to the left. Another person asked to count a metronome’s clicks exhibited minute hand movements that matched the rhythm. And when instructed to think about an object he hid, a person slowly moved a hand in the direction of that object. In each case, the person was not conscious of performing the actions at issue.

Utilization behavior, an interesting phenomenon, is displayed by people who suffer from frontal lobe damage of a certain kind (Wegner, 2002, p. 122). For example, one such person whose hands were touched with several pairs of eyeglasses over a brief period of time, put them all on and wound up wearing several pairs at once.

There are experimental situations in which participants are led to believe (to some degree) that they intentionally did things they did not in fact do. In a much-discussed study (Wegner and Wheatley, 1999), a confederate and a participant, both of whom are wearing headphones, jointly operate a computer mouse on which a small square board is mounted (p. 487). A computer monitor displays about fifty small objects, and the mouse controls the movement of a cursor over the display. Participants are asked how much they “intended” to make the cursor stop on an image (p. 488). On average, they give a higher “intended” rating to stops made on images whose names they heard shortly before the stop, even though the stop is produced by the confederate. (See Malle, 2006, pp. 223–224 for an instructive critique of this study.)

Findings and studies such as the ones I have described in this section have been offered in support of the contention that actions never have conscious proximal intentions or their physical correlates among their causes. Readers will recall that this is the thesis of *scientific epiphenomenalism* about conscious proximal intentions. Now, these findings and studies provide evidence that people sometimes perform actions of which they are not conscious, sometimes do things for no good reason, and sometimes believe (to some degree) that they intentionally did things they did not actually do. But how does Wegner get from evidence of the kind I described to the conclusion that conscious proximal intentions and their physical correlates are *never* among the causes of corresponding actions? That is, how does he get from this evidence to scientific epiphenomenalism about conscious proximal intentions?

Wegner (2002, p. 144) writes: “it has to be one way or the other. Either the automatisms are oddities against the general backdrop of conscious behavior causation in everyday life, or we must turn everything around quite radically and begin to think that behavior that occurs *with* a sense of will is somehow the odd case, an add-on to a more basic underlying system”. If it has to be one way or the other, then all actions have to be caused in the same basic way. So, Wegner thinks, if some actions are produced by automatic mechanisms rather than by conscious intentions or their physical correlates, then all of them are (perhaps with the exception of “the odd case”).

Are all actions caused in basically the same way? That depends on how we read “basically the same way.” If the claim is simply that all actions have brain events among their causes, then, obviously, the claim is true. However, this plainly leaves it open that some of the brain events that are among the causes of some actions are physical correlates of conscious intentions to perform actions of those kinds. It leaves the falsity of scientific epiphenomenalism about conscious intentions wide open. Of course, Wegner has something much more specific in mind – namely, that just as people who unwittingly make tiny hand movements in response to the clicks of a metronome are caused to do so by automatic processes of which they are unaware, all actions are caused by, and only by, such processes (perhaps with some odd exceptions).

Wegner’s (2002, p. 144) argument hinges on the idea that “it has to be one way or the other” – either unconscious automatic processes produce all of our actions or “conscious will” does it all. This bold formulation of the idea raises some questions. Is it true that it all has to be one way or the other? Might conscious proximal decisions or intentions or their physical correlates sometimes benefit from automatic mechanisms in the causation of actions? And what might constitute evidence that conscious proximal intentions or decisions or their physical correlates play a role in producing some actions?

Imagine a Libet-style study in which participants are asked to make *conscious proximal decisions* about when to flex a wrist and to flex in response to those decisions. Are they able to comply with this request, literally interpreted? Suppose that they do comply. Then, it seems, their conscious decisions or their physical correlates are indeed among the causes of their flexing actions. Such a decision or its physical correlate would be no less a cause of action than a go-signal is in a reaction time experiment. (Notice that I wrote “a cause,” not “the cause.”) Nothing in the data Wegner reports warrants the assertion that conscious proximal decisions (or their physical correlates), unlike go-signals, are never among the causes of associated actions.

A proponent of scientific epiphenomenalism about conscious decisions may contend that the participants in the experiment I imagined would have flexed even if they had *unconsciously* decided (or intended) to flex and that the conscious decisions and their physical correlates therefore made no causal contribution to the flexes. (Wegner himself cannot offer this reply, given his contention that all intentions are conscious. See Mele, 2009, chs. 2 and 5 for discussion of Wegner’s conception of intentions.) This contention is seriously misguided. It implicitly appeals to the following principle: If y would have happened even if x had not happened, then x is not among the causes of y. And this principle is false. Billy’s sister drove him to work today, and Billy arrived there at noon. What Billy’s sister did was a cause of Billy’s arriving at work when he did. And this is so even though, if Billy’s sister had not driven him to work, Billy’s brother would have done so and delivered him there at noon.

Another tack a proponent of scientific epiphenomenalism about conscious decisions may take is to claim that, in the imagined experiment, the participants’ conscious decisions were not among the causes of their flexes because the decisions themselves were caused by unconscious processes. However, a reader who finds this claim appealing has lost sight of the moral of my firecracker analogy. The fact that x has a cause is utterly compatible with x being among the causes of y.

I had originally planned to include a brief section on hard evidence that conscious intentions (or their physical correlates) sometimes *are* among the causes of corresponding actions. This evidence comes from research on implementation intentions (Gollwitzer, 1999, 2014; Gollwitzer and Sheeran, 2006). However, I found that I had nothing noteworthy to add to my discussion of the evidence in chapter 7 of Mele (2009). So I simply direct interested readers to that chapter. The evidence discussed there is impressive.

I conclude this section with its main moral. The second line of argument for scientific epiphenomenalism about conscious intentions depends on Wegner’s assumption that either “conscious will” produces all of our actions or unconscious automatic processes do all the work (perhaps with some unusual exceptions), and there is no good reason to accept that assumption. For all anyone has shown, many actions – including many actions in the moral sphere – may be products of a combination of conscious intentions (or their physical correlates) and unconscious processes.

## Conclusion

What I called scientific epiphenomenalism is advanced as a serious challenge to free will – and, by extension, moral responsibility. In this article I examined two main lines of argument for scientific epiphenomenalism. The first line is based on Libet’s studies, discussed in section 2. There I explained why a certain data-based argument for the nonexistence of free will that applies also to moral responsibility is unsuccessful. In the course of so doing, I highlighted some important differences between the decisions that are supposedly made in the experiments at issue and decisions made when moral considerations are in play. The differences are such that a generalization from alleged findings about the decisions studied in Libet-style experiments to a conclusion about all decisions is unwarranted.

The second line of argument is based on a mixed bag of findings reported by Wegner, examined in section 3. As I explained, that line of argument hinges on an assumption that there is no good reason to accept – the assumption that “it has to be one way or the other” (Wegner, 2002, p. 144) or, more fully, that either all of our actions are produced by, and only by, unconscious automatic processes (once again, perhaps with some odd exceptions) or “conscious will” does all the work.

Roughly speaking, what it is for an intention to be effective is for it to be among the causes of the agent’s performance of an action of the kind intended.^6^ To accommodate metaphysically minded readers who may maintain that physical correlates of intentions, but not intentions themselves, play the causal role, it may be said that what it is for an intention to be effective is for the intention to be such that either it or its physical correlate is among the causes of the agent’s performance of an action of the kind intended. On metaphysical grounds, some readers will deem this construal of effective intentions too permissive. I remind such readers that scientific epiphenomenalism asserts that, even on this metaphysically permissive construal, there are no effective intentions. The two lines of argument examined here pose at best an illusory threat to the existence of effective intentions (so construed), including effective *conscious* intentions. The same goes for free will and moral responsibility.

Why have I focused here on scientific epiphenomenalism rather than philosophical epiphenomenalism? Mainly, because, even if all relevant scientific facts were discovered, and even if they proved that the physical correlates of conscious intentions (that is, of the intentions *and* consciousness of them, as I said earlier) often play a causal role in producing corresponding actions, it would still be open to metaphysicians to argue that this is not sufficient for the truth of the claim that conscious intentions play a causal role in producing these actions. What would have to be added to the mix to achieve sufficiency is the truth of some metaphysical thesis or other that is scientifically untestable. Because this article’s topic is *scientific* arguments for certain theses, I chose to focus on matters that are, in principle, scientifically testable. The scientific work and arguments I have discussed here leave ample room for moral responsibility and free will.^7^

## Author Contributions

The author confirms being the sole contributor of this work and has approved it for publication.

## Conflict of Interest Statement

The author declares that the research was conducted in the absence of any commercial or financial relationships that could be construed as a potential conflict of interest.

## Footnotes

1 A referee asked for other findings about Philomena. Here are the figures for agreement: When she irritated the cyclist, Filomena intended to do that. Agree 5.44%. When she released a lot of exhaust fumes, Filomena intended to do that. Agree 7.42%. Filomena intentionally released a lot of exhaust fumes. Agree 7.42%.

2 Kim (2003) is an excellent introduction to supervenience.

3 For an accessible critical review of various relevant philosophical positions that highlights the metaphysical nature of the debate, see Jackson (2000).

4 This assumption has been questioned (Warfield, 2003). For discussion, see Mele (2017), ch. 5.

5 Libet maintains that although no decisions to *act* are freely made, decisions not to act on an intention to do something – decisions that *veto* intentions to do things – may be freely made (2004, pp. 137–149). For discussion of Libet on vetoing, see Mele (2009), pp. 61–61 and 69–86.

6 For readers who are familiar with the philosophical literature on causal deviance, I observe that an intention may be effective either in a deviant or in a non-deviant way. For readers who are unfamiliar with this literature, I offer an example. Ann and Bob are standing at entrances to opposite sides of a building. To unlock the door, they need to punch in a certain five digit code. Ann knows the code and Bob does not; but Bob believes, incorrectly, that any five digit code will work. Both intend to unlock the door in front of them, and both act accordingly. As luck would have it, Bob happens to type in the correct code. His unlocking the door is too lucky to count as intentional; and his intention to unlock the door is effective, but in a deviant way. Ann’s intention is non-deviantly effective.

7 I am grateful to Mario De Caro, Sofia Bonicalzi, James Moore, and an anonymous referee for comments on a draft of this article.

## References

[B1] Fried I. Mukamel R. Kreiman G. (2011). Internally generated preactivation of single neurons in human medial frontal cortex predicts volition. *Neuron* 69 548–562. 10.1016/j.neuron.2010.11.045 21315264 PMC3052770

[B2] Gollwitzer P. (1999). Implementation intentions. *Am. Psychol.* 54 493–503. 10.1037/0003-066X.54.7.493

[B3] Gollwitzer P. (2014). Weakness of the will: is a quick fix possible? *Motiv. Emot.* 38 305–322. 10.1007/s11031-014-9416-3

[B4] Gollwitzer P. Sheeran P. (2006). Implementation intentions and goal achievement: a meta-analysis of effects and processes. *Adv. Exp. Soc. Psychol.* 38 69–119. 10.1016/S0065-2601(06)38002-1 18096108

[B5] Haggard P. Mele A. O’Connor T. Vohs K. (2015). “Free will lexicon,” in *Surrounding Free Will*, ed. Mele A. (New York, NY: Oxford University Press), 319–326.

[B6] Haggard P. Clark S. (2003). Intentional action: conscious experience and neural prediction. *Conscious. Cogn.* 12 695–707. 10.1016/S1053-8100(03)00052-7 14656511

[B7] Jackson F. (2000). Psychological explanation and implicit theory. *Philos. Explor.* 3 83–95. 10.1080/13869790008520982

[B8] Kim J. (2003). “Supervenience, emergence, realization, reduction,” in *The Oxford Handbook of Metaphysics*, eds Loux M. Zimmerman D. (New York, NY: Oxford University Press), 556–584.

[B9] Libet B. (1985). Unconscious cerebral initiative and the role of conscious will in voluntary action. *Behav. Brain Sci.* 8 529–566. 10.1017/S0140525X00044903

[B10] Libet B. (2001). Consciousness, free action and the brain. *J. Conscious. Stud.* 8 59–65.

[B11] Libet B. (2004). *Mind Time.* Cambridge, MA: Harvard University Press.

[B12] Libet B. Wright E. Gleason C. (1982). Readiness potentials preceding unrestricted ‘spontaneous’ vs. pre-planned voluntary acts. *Electroencephalogr. Clin. Neurophysiol.* 54 322–335. 10.1016/0013-4694(82)90181-X 6179759

[B13] Malle B. (2006). “Of windmills and straw men: folk assumptions of mind and action,” in *Does Consciousness Cause Behavior? An Investigation of the Nature of Volition*, eds Pockett S. Banks W. Gallagher S. (Cambridge, MA: MIT Press), 207–231.

[B14] Mele A. (2003). *Motivation and Agency.* New York, NY: Oxford University Press 10.1093/019515617X.001.0001

[B15] Mele A. (2009). *Effective Intentions.* New York, NY: Oxford University Press 10.1093/acprof:oso/9780195384260.001.0001

[B16] Mele A. (2013a). Free will and neuroscience. *Philos. Exch.* 43 1–17.

[B17] Mele A. (2013b). Unconscious decisions and free will. *Philos. Psychol.* 26 777–789. 10.1080/09515089.2012.724395

[B18] Mele A. (2017). *Aspects of Agency: Decisions, Explanations, Abilities, and Free Will.* New York, NY: Oxford University Press 10.1093/acprof:oso/9780190659974.001.0001

[B19] Papineau D. (2009). “The causal closure of the physical and naturalism,” in *The Oxford Handbook of Philosophy of Mind*, eds McLaughlin B. Beckermann A. Walter S. (New York, NY: Oxford University Press),53–65.

[B20] Pockett S. (2006). “The neuroscience of movement,” in *Does Consciousness Cause Behavior? An Investigation of the Nature of Volition*, eds Pockett S. Banks W. Gallagher S. (Cambridge, MA: MIT Press), 9–24. 10.7551/mitpress/9780262162371.001.0001

[B21] Pockett S. Banks W. Gallagher S. (eds). (2006). *Does Consciousness Cause Behavior? An Investigation of the Nature of Volition.* Cambridge, MA: MIT Press 10.7551/mitpress/9780262162371.001.0001

[B22] Roediger H. Goode M. Zaromb F. (2008). “Free will and the control of action,” in *Are We Free? Psychology and Free Will*, eds Baer J. Kaufman J. Baumeister R. (New York, NY: Oxford University Press), 205–225. 10.1093/acprof:oso/9780195189636.003.0010

[B23] Soon C. S. Brass M. Heinze H. J. Haynes J. D. (2008). Unconscious determinants of free decisions in the human brain. *Nat. Neurosci.* 11 543–545. 10.1038/nn.2112 18408715

[B24] Warfield T. (2003). “Compatibilism and incompatibilism: some arguments,” in *The Oxford Handbook of Metaphysics*, eds Loux M. Zimmerman D. (New York, NY: Oxford University Press), 613–630.

[B25] Wegner D. (2002). *The Illusion of Conscious Will.* Cambridge, MA: MIT Press.

[B26] Wegner D. (2004). Précis of the illusion of conscious will. *Behav. Brain Sci.* 27 649–659. 10.1017/S0140525X04000159 15895616

[B27] Wegner D. (2008). “Self is magic,” in *Are We Free? Psychology and Free Will*, eds Baer J. Kaufman J. Baumeister R. (New York, NY: Oxford University Press), 226–247. 10.1093/acprof:oso/9780195189636.003.0011

[B28] Wegner D. Wheatley T. (1999). Apparent mental causation: sources of the experience of will. *Am. Psychol.* 54 480–491. 10.1037/0003-066X.54.7.480 10424155

[B29] Zimmerman D. (2006). “Dualism in the philosophy of mind,” in *Encyclopedia of Philosophy*, 2nd Edn Vol. 3 ed. Borchert D. (Detroit, MI: Thomson Gale), 113–122.

